# Real‐time fMRI neurofeedback normalizes hippocampal hyperactivity and improves memory performance in preclinical Alzheimer's disease

**DOI:** 10.1002/alz70856_100817

**Published:** 2025-12-25

**Authors:** Katharina Klink, Jessica Peter

**Affiliations:** ^1^ University of Bern, Bern, Switzerland

## Abstract

**Background:**

Several fMRI studies in patients with Mild Cognitive Impairment (MCI) found hippocampal hyperactivity during a pattern separation task, which was associated with accelerated accumulation of Alzheimer's disease pathology, faster disease progression and more pronounced cognitive decline. We tested whether real‐time fMRI neurofeedback, during which individuals learn to regulate region specific brain activity, can reduce hippocampal hyperactivity and thereby improve pattern separation performance.

**Methods:**

Seventy‐eight participants, categorized by clinical diagnosis (MCI or healthy), blood‐based biomarker (pTau181 above or below cutoff), or amyloid positivity risk (high or low), were enrolled. Participants learned how to downregulate activity in their own hippocampus or in a control region (two times, for 1 hour each). One week later, we re‐assessed hippocampal activity and pattern separation performance.

**Results:**

Downregulation of hippocampal activity during neurofeedback led to significantly reduced hippocampal activity and significantly improved pattern separation performance one week later. Downregulation of activity in a control region had no significant effect on hippocampal activity and only minor effects on pattern separation performance. These findings were consistent across subgroups based on clinical diagnosis, amyloid positivity risk, and biomarker status.

**Conclusion:**

Our results indicate that real‐time fMRI neurofeedback can effectively downregulate hippocampal activity and improve pattern separation performance in individuals at risk of developing Alzheimer's disease. This approach holds promise as a potential therapeutic tool to address hippocampal dysfunction in early Alzheimer's disease.

